# Prevalence of Sexual Dysfunction among Infertile Women in Iran: A Systematic Review and Meta-analysis

**DOI:** 10.22074/ijfs.2019.5395

**Published:** 2018-10-02

**Authors:** Reza Omani-Samani, Payam Amini, Behnaz Navid, Mahdi Sepidarkish, Saman Maroufizadeh, Amir Almasi-Hashiani

**Affiliations:** Department of Epidemiology and Reproductive Health, Reproductive Epidemiology Research Centre, Royan Institute for Reproductive Biomedicine, ACECR, Tehran, Iran

**Keywords:** Female, Infertility, Iran, Prevalence

## Abstract

Infertile women are at a higher risk of sexual dysfunction compared to fertile women. Infertility is a major source of stress, anxiety, and depression, which strongly affects sexual health. The aim of this study is to estimate the prevalence of female sexual dysfunction (FSD) among infertile Iranian women. We searched the main international databases (Web of Science, PubMed, Medline, and Scopus) and national databases (Scientific Information Database, Magiran, and IranMedex) from their inception until April, 2017. Due to heterogeneity between the studies, the extracted data were pooled using a random-effects model by Stata software. Out of 313 retrieved studies, we included 18 studies of 3419 infertile women in the meta-analysis. The pooled prevalence of FSD was 64.3% [95% confidence interval (CI): 53.3-75.3]. Our findings revealed that sexual desire (59.9%, 95% CI: 38.7-81.2) was the most prevalent disorder and vaginismus (19.2%, 95% CI: 11.3-27.2) was the least prevalent among infertile women. The results of our meta- analysis suggested that more than 64% of infertile Iranian women reported sexual dysfunction, which was meaning- fully high. This study also showed that sexual desire was significantly more common than other sexual dysfunction dimensions and the prevalence of vaginismus was the least common.

## Introduction

The estimated prevalence of infertility is approximately 9% worldwide (1). Infertility has negative effects on emotional health, quality of life, and a couple′s sexual relationship (2, 3). Infertility has a greater impact of psychosexual behaviour in women than men (4). Sexual function is one of the most important components of quality of life and social health (5). The estimated prevalence of sexual problems in Iranian women is 31.5%, and for Iranian men, it is 18.8% (6). Many studies report that sexual dysfunction is more common among infertile women (7, 8).

Sexual dysfunction has several domains. The Diagnostic and Statistical Manual of Mental Disorders (DSM-5) lists the types of sexual dysfunctions in females as female sexual interest/arousal disorder, female orgasmic disorder, and genito-pelvic pain/penetration disorder (9). The Female Sexual Function Index (FSFI) contains four domains: sexual arousal, orgasm, satisfaction, and pain (10).

There is an association between sexual dysfunction and infertility (11). Sexual dysfunction may cause difficulties in sexual function during attempts to conceive (12). In order to perform the diagnostic assessment and sexual dysfunction therapy in infertile women, it is necessary to specify the prevalence of these disorders. The prevalence of sexual dysfunction varies across populations and is affected by medical, psychological, socioeconomic, cultural, and ethnic factors (13).

Many studies conducted in Iran to evaluate the prevalence of sexual dysfunction among infertile women have reported various findings (14-17). Thus, we conducted this meta-analysis to estimate the prevalence rate of sexual dysfunction in infertile Iranian women.

## Materials and Methods

### Search strategy

Royan Institute approved this systematic review and meta-analysis (code: 95000051). The authors followed the Preferred Reporting Items for Systematic Reviews and Meta-Analyses (PRISMA) checklist to perform this meta-analysis (18). The authors searched for the prevalence of female sexual dysfunction (FSD) in infertile Iranian women. We searched published literature in the international (Web of Science, PubMed, Scopus) and national (Magiran, SID, and IranMedex) electronic databases from their inception until April 2017. Key words used for the search included “sexual problem”, “sexual disability”, “sexual dysfunction”, “sexual dysfunction, physiological,”, “sexual problems”, “sexual pain”, “orgasm”, “lubrication”, “sexual excitement”, ”sexual desire”, “dyspareunia”, “vaginismus”, “Iran”, “infertility”, “infertility, female”, “cross-sectional study”, “prevalence study” and “prevalence”. No time restriction was applied to the searches and we included both Farsi and English languages in the study. In addition to the mentioned databases, the grey literatures were searched using Google Scholar for the possibility of missed papers as recommended by Haddaway et al. (19). We also checked the reference lists of the included articles for additional potentially applicable papers.

### Inclusion and exclusion criteria

Studies with the estimated prevalence rates of FSD, observational studies, studies in Farsi and English languages, and those without any restricted published date were included in this study. Excluded from this meta-analysis were interventional studies, repeated or duplicated studies, and studies with no relevant reported data.

### Data extraction and quality assessment

In this meta-analysis, 2 authors (AAH and SM) separately extracted the required data from the included studies. Data extracted were: first Authors' name, year of publication, place of study, published year of study, mean age, infertility year, sample size, type of questionnaire, and the prevalence estimate of FSD and its dimensions. Then, 2 reviewers (AAH and MS) independently performed the quality assessment based on our modified STROBE checklist (20). The quality of the papers was low (22.22%), moderate (61.11%), and high (16.67%). This checklist contained sample size, sampling method, analysis, generalizability, quality of results reported, and study design.

### Statistical analysis

The pooled prevalence was estimated by the “metan” command in Stata. Statistical heterogeneity between studies was checked by the Cochrane Q test and I2 statistics. Because of low primary studies, for the Cochrane Q test, we considered a P<0.10 to be statistically significant. An value of 25% indicated low heterogeneity, 50% was moderate, and 75% indicated high heterogeneity (21).

The outcome measure of study was prevalence of sexual dysfunction in infertile women. In terms of the outstanding heterogeneity among the studies, we applied a random effect model to pool the primary prevalence rates. To explain the sources of between-study heterogeneity, meta-regression was performed for the year of the study, the sample size, and type of questionnaire. By running the “metainf” command, we conducted sensitivity analyses by excluding each study from the analysis to examine the influence of each study on the pooled estimate. The Funnel plot, Begg's rank correlation, and Egger's weighted regression tests were used to assess publication bias (22, 23). The level of significance in these tests was less than 0.10 because of the statistical power. Finally, cumulative meta-analysis was performed to investigate whether the amount of prevalence changed noticeably over time (“metacum” command). All statistical analyses were performed using Stata version 14.0 (Stata Corp., College Station, TX, USA).

## Results

### Study selection

**Fig.1:**
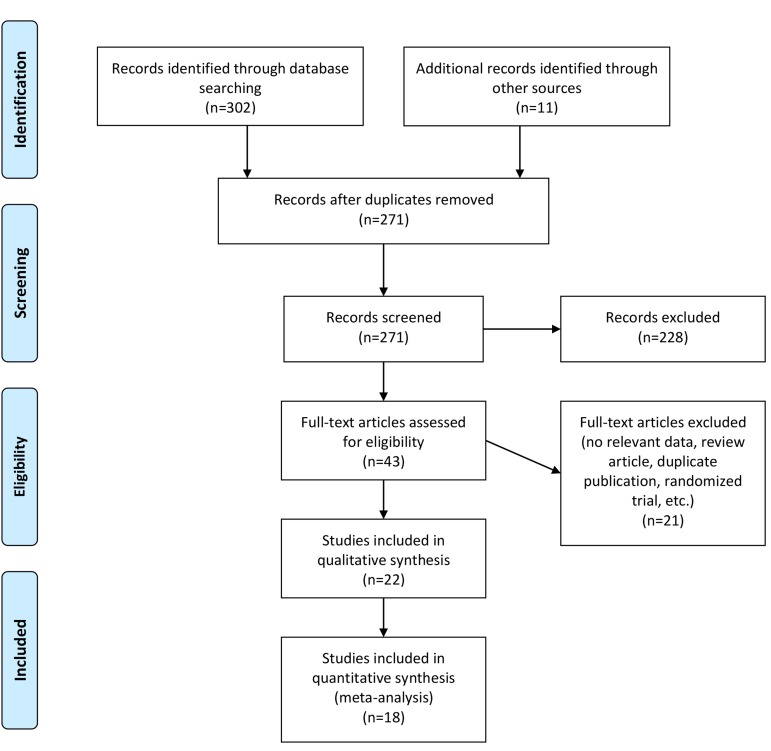
Flow diagram of the literature search for studies included in the meta-analysis.

The details of the study selection method are shown in Figure 1. We identified a total of 313 relevant papers; after removal of the duplicates, 271 papers remained. After screening the titles and abstracts, we disqualified 228 papers, and resumed the full texts for 43 relevant papers. Next, we excluded all non-eligible studies, which left a total of 18 cross-sectional or case control studies based on the inclusion criteria for the meta-analysis.

### Study characteristics

The majority (two-thirds) of the studies used the FSFI questionnaire to assess the prevalence of FSD. The lowest prevalence of FSD among infertile women was 46.6%, whereas the highest prevalence of FSD was 87.1%. These studies were published between 2001 and 2017 and had a diverse sample size that ranged from 30 to 604 cases, with a total of 3419 infertile women. Additional information about each primary study included in this analysis is shown in Table 1.

**Table 1:** Characteristics of the primary studies included in the meta-analysis


ID	Author	Yearpublished	City	Samplesize	Age (Y)(mean + SD)	Mean years of infertility (mean + SD)	Questionnaire	Sampling method	Quality assessment

1	Sargolzaee et al. (24)	2001	Mashhad	30	25.77 ± 5.08	4.2 ± 3.09	GSF	Random	Moderate
2	Besharat and Hoseinzadeh Bazargani (25)	2006	Tehran	45	28.8 ± 4.68	NA	Golombok-Rust	Convenience	Low
3	Tayebi and Yassini Addakani (26)	2007	Yazd	300	27.93 ± 4.8	5.42 ± 3.2	NR	Volunteer	Moderate
4	Khademi et al. (27)	2008	Tehran	100	26.9 ± 5	5.3 ± 3.7	SFQ	Volunteer	Moderate
5	Fahami et al. (28)	2009	Isfahan	140	29 ± 5.5	6.5 ± 5.2	FSFI	Convenience	Moderate
6	Pakpour et al. (8)	2012	5 cities	604	30 ± 7.8	NA	FSFI	Convenience	High
7	Aghamohammadian Sharbaf (29)	2014	Mashhad	200	28.8 ± 6.2	NA	FSFI	Convenience	Moderate
8	Basirat et al. (30)	2014	Babol	208	27.85 ± 5.7	NA	FSFI	NA	High
9	Davari Tanha et al. (11)	2014	Tehran	320	29.66	NA	FSFI	NA	Moderate
10	Hashemi et al. (31)	2014	Tehran	128	30.9 ± 4.9	NA	FSFI	NA	Moderate
11	Jamali et al. (32)	2014	Jahrom	100	28.56 ± 5.72	NA	FSFI	Random	High
12	Jamali et al. (33)	2014	Jahrom	502	30.95 ± 6.80	NA	FSFI	Convenience	Moderate
13	Karamidehkordi and Roudsari (34)	2014	Mashhad	130	27 ± 4.58	NA	FSFI	Convenience	Low
14	Alirezaee et al. (35)	2014	Mashhad	85	NA	NA	FSFI	Convenience	Low
15	Bakhtiari et al. (36)	2016	Babol	236	26.1 ± 5.3	60.2 ± 8.4 months	DSM	Convenience	Moderate
16	Mirblouk et al. (37)	2016	Guilan	147	31.66 ± 6.8	NA	FSFI	NA	Moderate
17	Zare et al. (38)	2016	Mashhad	110	29.2 ± 4.9	4.85 ± 3.53	Golombok-Rust	Convenience	Moderate
18	Billar et al. (39)	2017	2 cities	34	42	NA	FSFI	Convenience	Low


GSF; Global Sexual Functioning Scale, SFQ; Sexual Function Questionnaire, FSFI; Female Sexual Function Index, NR; Not reported, and DSM; The diagnostic and statistical manual of mental disorders.

### Evaluation of heterogeneity and meta-analysis

**Fig.2:**
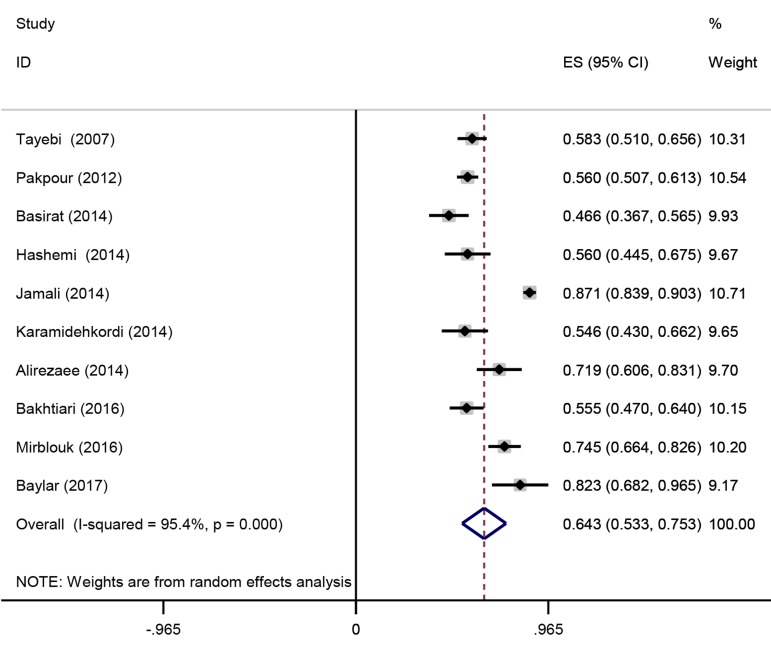
Forest plot that shows the prevalence of female sexual dysfunction (FSD) among infertile Iranian women.

The results of Cochran’s Q test and I2 statistics displayed considerable heterogeneity among the primary studies included for FSD (Q=194.04, P=0.0001 and I2: 95.4%); thus, we used the random effects model for analysis. The pooled prevalence of FSD was 64.3% (95% CI: 53.3-75.3). As shown in Figure 2, the lowest prevalence of FSD was reported by Basirat et al. (30) in Babol, Northern Iran (46.6%, 95% CI: 36.7%-56.5%) and Jamali et al. (33) reported the highest prevalence in Jahrom, Southern Iran (87.1%, 95% CI: 83.9%-90.3%).

The pooled estimated prevalence of different dimensions of sexual dysfunction that included sexual desire, sexual excitement, orgasm, dyspareunia and vaginismus is presented in Table 2. The results showed that the most prevalent sexual disorder was related to sexual desire (59.9%; 95% CI: 38.7-81.2) and the least prevalent was vaginismus (19.2%, 95% CI: 11.3-27.2).

**Table 2:** The pooled estimated prevalence of different dimensions of sexual dysfunction


Disorder	Number of included studies	Pooled estimated (%)	95% CI	I^2^ (%)

Sexual desire	8	59.9	38.7-81.2	99.2
Sexual excitement	4	52.3	29.6-75.0	96.9
Orgasm	7	53.8	27.9-79.7	99.4
Dyspareunia	6	52.9	29.4-76.4	98.8
Vaginismus	2	19.2	11.3-27.2	82.6


CI; Confidence interval and I2; I square.

### Publication bias

We used Begg’s test to assess for probable publication bias of FSD prevalence. The results showed no evidence of any publication bias (P=0.325).

### Meta-regression

In order to assess the sources of heterogeneity, we included 4 variables in a univariate meta-regression. The results suggested that the study sample size (P=0.992), date (P=0.366), type of questionnaire (P=0.418), and age (P=0.070) were not accountable for the heterogeneity in the FSD prevalence. Therefore, we used the random effect model because of the presence of heterogeneity between studies.

### Sensitivity analysis and cumulative meta-analysis

In order to calculate the influence of each primary study, a sensitivity analysis was performed by removing each study from the analysis and calculating the point estimates. The results of the sensitivity analysis (Table 3) showed that after removal of the individual studies, the pooled prevalence of FSD ranged from 61.2%, after excluding Jamali et al. (33) to 66.2% after excluding Basirat et al. (30).

**Table 3:** Sensitivity analysis to estimate the pooled prevalence by removal of each individual study


Study omitted	Pooled prevalence	95% CI	

Basirat et al. (30)	0.662	0.774	0.550
Karamidehkordi and Roudsari (34)	0.653	0.769	0.537
Bakhtiari et al. (36)	0.653	0.770	0.536
Hashemi et al. (31)	0.652	0.768	0.535
Pakpour et al. (8)	0.653	0.770	0.535
Tayebi and Yassini Addakani (26)	0.650	0.769	0.530
Alirezaee et al. (35)	0.635	0.754	0.515
Mirblouk et al. (37)	0.631	0.754	0.509
Billar et al. (39)	0.625	0.742	0.508
Jamali et al. (33)	0.612	0.678	0.546


**Fig.3:**
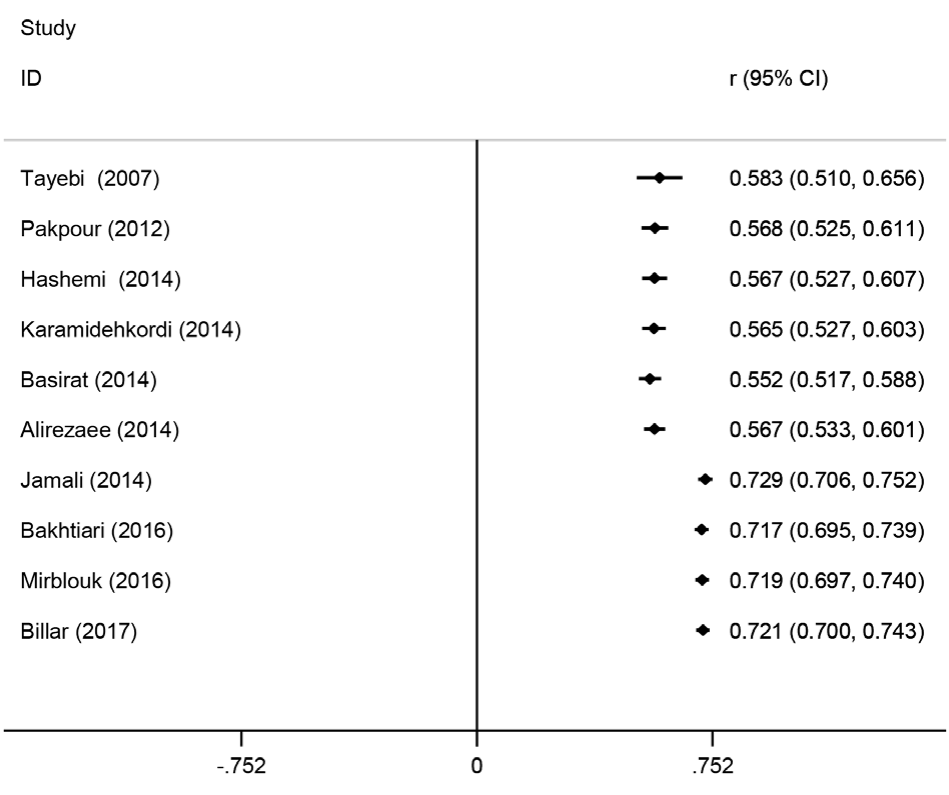
Cumulative meta-analysis of female sexual dysfunction (FSD) by sorting the studies based on publication time.

After sorting the studies based on publication year, the cumulative meta-analysis showed that the overall prevalence estimate was not constant over time; rather there was an increase after 2014 (Fig .3).

## Discussion

The tendency of having sexual intercourse is strongly affected by pregnancy, which results in a low FSD score. It is well-known that infertile women are at a higher risk of sexual dysfunction compared to fertile women (33, 40, 41). Infertility is a major source of stress, anxiety and depression, which strongly affects sexual health. It has been shown that sexual dysfunction simultaneously compounds the disappointment of childlessness and the distress of medical treatment among infertile patients (42). However, sex is less defined as a loving act and considered more of a clinical tool among infertile couples (14). Our study has revealed that 64% of infertile women in Iran have sexual dysfunction. The studies were published with different sample sizes over a 17-year period. However, the current study showed that the diversity in the FSD prevalence was not affected by sample size, date, age, and type of questionnaires in Iran. This study also demonstrated that vaginismus was significantly less prevalent than other sexual dysfunction dimensions. Regarding the vast spectrum of vaginismus, women might not be aware of their disorder, which might lead to a low prevalence rate. Psychological variables are the most responsible factors for vaginismus (43).

Although the prevalence of sexual desire was higher than the other dimensions, dyspareunia, orgasm, and sexual excitement did not considerably differ in prevalence ratios. Based on the results of our meta-analysis, the prevalence of FSD among infertile women in Iran was noticeable. This might be due the adverse consequences of infertility such as personal and marital distress, depression, anxiety, reduction in self-esteem, and greater risk of psychological pressure that strongly contributes to sexual dysfunction in women (44). It has been demonstrated that both of the diagnosis of infertility and the treatments affect FSD (41). Some local and cultural aspects could reduce the amount of sexual functioning among Iranian infertile women such as lack of sexual knowledge and poor emotional relationship, the presence of economic problems, and pregnancy as the only point for sexual function.

Keskin et al. (45) found that 64.8% of women with primary infertility and 76.5% of those with secondary infertility had sexual dysfunction. Aggarwal et al. (4) reported that 63.67% of infertile women had FSD. Potential factors such as kidney failure, multiple sclerosis, heart disease and bladder problems, hormonal dysfunctions, and social and psychological problems might be responsible (8, 46). In comparison to the Middle East, the FSD prevalence rate is relatively higher in Iran (47). However, the difference is not considerable, which might be due to the similarities in culture and the same amount of development. However, the respondents were self-reporting in Iranian studies. According to cultural conditions, patients might not provide the exact responses to the questions and there might be biases in the prevalence rate.

There may be a two-way relationship between infertility and sexual dysfunction. Infertility changes sexual feelings and sexual dysfunction may result in infertility. However, numerous potential factors cause the increase in FSD prevalence among infertile women and include involuntary childlessness, woman’s age, husband’s age, the lack of knowledge about marital issues, lack of training in the society, socio-economic status, infertility characteristics, the relationship with partner, duration of marriage, medical problems, depression, anxiety, loss of self-esteem, menopausal status, history of previous infertility treatment, income level, lower educational level, frequency of intercourse, and higher partner education (8, 45, 48-53).

Infertility affects the dimensions of sexual dysfunction (2, 37, 44, 53). In 2 different studies conducted by Keskin et al. (45) in Turkey and Pakpour et al. (8) in Iran, the researchers reported that the prevalence of sexual desire, orgasm, and satisfaction decreased among women with secondary infertility compared to those with primary infertility. Iris et al. (2) investigated the effects of infertility and infertility duration on female sexual function. They demonstrated that the mean score of all sexual functions such as desire, arousal, lubrication, orgasm, sexual satisfaction, and pain, as well as the total score decreased over time. The similarity of the prevalence ratios among the FSD dimensions in Iran might be due to the similarity in their risk factors. These potential factors could explain the difference in prevalence ratios across countries. Berger et al. (54) assessed the association between infertility and sexual dysfunction in men and women. They indicated that desire was strongly associated with problems in achieving pregnancy and infertility. These researchers introduced sexual dysfunction as a complex issue among couples with infertility and suggested that health policy makers should utilize appropriate medical therapy and psychosocial tools for infertile couples.

However, the power of statistical tools that has identified the heterogeneity in the studied meta-analysis differs according to the sample size of the studies as well as the number of included studies. The chi-square test is strongly affected by these limitations, such that a non-significant result must not be taken as evidence of lack of heterogeneity. On the other hand, the power of the chi-square test is high when many studies are included in a meta-analysis. The I2 value depends on the magnitude of the prevalence ratios (55). In our meta-analysis, the result of chi-square test has been confirmed by the I2 test, which addressed considerable heterogeneity among the reported prevalence ratios of the included studies. These studies were conducted in different regions of the country. The heterogeneity might be due to the diversities in the ethnic and cultural conditions, uneven development regions and disparity in the amount of knowledge, particularly about sexual performance.

Limitations in this study included the use of different questionnaires with different scoring methods to assess the prevalence of sexual dysfunction; therefore, we did not pool all of the scores in a continuous scale. In some studies, the scores of the questionnaires (in a continuous scale) was reported, whereas in other studies, the prevalence of FSD (in a categorized scale) was reported. There were different cut-offs for the questionnaires. For example, the point at which a woman was classified as having a sexual dysfunction or not might have been used in the studies. However, we ignored this issue and pooled the reported prevalence rate.

## Conclusion

The results of current meta-analysis discovered that prevalence of FSD in infertile Iranian women was considerable. More than 64% of these women had sexual dysfunction. This study also showed that sexual desire was significantly more common than other sexual dysfunction dimensions and that the prevalence of vaginismus was less than the other dimensions.
